# Scrub Typhus and Molecular Characterization of *Orientia tsutsugamushi* from Central Nepal

**DOI:** 10.3390/pathogens10040422

**Published:** 2021-04-01

**Authors:** Rajendra Gautam, Keshab Parajuli, Mythili Tadepalli, Stephen Graves, John Stenos, Jeevan Bahadur Sherchand

**Affiliations:** 1 Department of Microbiology, Maharajgunj Medical Campus, Institute of Medicine, Kathmandu 44600, Nepal; gautamrajen@gmail.com (R.G.); drkparajuli@iom.edu.np (K.P.); jeevanbsherchand@gmail.com (J.B.S.); 2 Australian Rickettsial Reference Laboratory, Geelong, VIC 3220, Australia; mythili.tadepalli@barwonhealth.org.au (M.T.); graves.rickettsia@gmail.com (S.G.)

**Keywords:** vector, molecular typing, 56 kDa, 47 kDa, qPCR

## Abstract

Scrub typhus is a vector-borne, acute febrile illness caused by *Orientia tsutsugamushi*. Scrub typhus continues to be an important but neglected tropical disease in Nepal. Information on this pathogen in Nepal is limited to serological surveys with little information available on molecular methods to detect *O. tsutsugamushi*. Limited information exists on the genetic diversity of this pathogen. A total of 282 blood samples were obtained from patients with suspected scrub typhus from central Nepal and 84 (30%) were positive for *O. tsutsugamushi* by 16S rRNA qPCR. Positive samples were further subjected to 56 kDa and 47 kDa molecular typing and molecularly compared to other *O. tsutsugamushi* strains. Phylogenetic analysis revealed that Nepalese *O. tsutsugamushi* strains largely cluster together and cluster away from other *O. tsutsugamushi* strains from Asia and elsewhere. One exception was the sample of Nepal_1, with its partial 56 kDa sequence clustering more closely with non-Nepalese *O. tsutsugamushi* 56 kDa sequences, potentially indicating that homologous recombination may influence the genetic diversity of strains in this region. Knowledge on the circulating strains in Nepal is important to the development of diagnostic tests and vaccines to support public health measures to control scrub typhus in this country.

## 1. Introduction

Scrub typhus is a vector-borne, acute febrile illness caused by *Orientia tsutsugamushi* and transmitted predominantly by mite larvae (“chiggers”) of the genus *Leptotrombidium* (usually species *deliense*) [1]. The rapid and accurate diagnosis of rickettsial illness is important for the appropriate antibiotic treatment. Traditionally, the diagnosis of scrub typhus is based on serological tests. Among the various serological tests, indirect immune fluorescence assay (IFA) is the most sensitive and specific test, and considered to be the gold standard test, but it is not usually positive in the acute phase of the disease as antibody levels become positive during convalescence [2]. The most sensitive test for the detection of *O. tsutsugamushi* is a culture, but it requires a BSL-3 laboratory and can take up to 60 days to yield a positive result, limiting its use in clinical diagnosis of this infectious disease [2]. As an alternative, polymerase chain reaction (PCR) is a highly sensitive and specific molecular detection method that can be used for the direct detection of *O. tsutsugamushi* DNA in blood and eschar samples [3]. However, it is not widely used in resource-limited settings.

*O. tsutsugamushi* strains can be differentiated serologically, primarily through immunoassays targeting the *O. tsutsugamushi* 56 kDa type-specific antigen. The 56 kDa type-specific antigen gene encodes a membrane protein, which accounts for 10–15% of the total protein of *O. tsutsugamushi,* and is highly immunogenic in humans [4]. This protein [5], along with others, including the *O. tsutsugamushi* 47 kDa antigen [6], has been the target of ongoing efforts to develop scrub typhus vaccines. To support efforts to determine the coverage of vaccines targeting these immunodominant *O. tsutsugamushi* proteins, but also to understand the broader genetic diversity of this intracellular pathogen, the genes encoding these proteins have been extensively studied. Molecular typing studies of the *O. tsutsugamushi* 56 kDa protein gene have revealed significant divergence among *O. tsutsugamushi* isolates of >80% nucleotide similarity [7,8,9]. In contrast, molecular typing of the *O. tsutsugamushi* 47 kDa protein gene revealed less diversity than that observed for the 56 kDa protein gene, with 96.7% to 100% sequence identity reported among a geographically diverse group of strains [6].

Scrub typhus is reported from several geographical areas of Nepal and accounts for 25%–40% of acute febrile illness [10,11]. Currently, there is a lack of information on the molecular detection and characterization of the strains of *O. tsutsugamushi* that occurs in Nepal. Hence, this study was conducted to initially detect *O. tsutsugamushi* and to molecularly characterize the strains in order to assist with diagnostic assay development.

## 2. Results

Rickettsial qPCR screening of 282 blood samples obtained from patients with suspected scrub typhus from central Nepal revealed an overall positivity of 29.8% (84/282).

Seventeen positive samples with relatively strong Ct values between 24 and 32 were subjected to conventional PCR amplification and sequencing of the 56 kDa and 47 kDa genes, obtaining partial sequences for both genes from six samples and a further four 47 kDa sequences from an additional four samples. The 10 samples from which 56 kDa and 47 kDa gene sequences were amplified were named Nepal_1–10.

**Figure 1:**
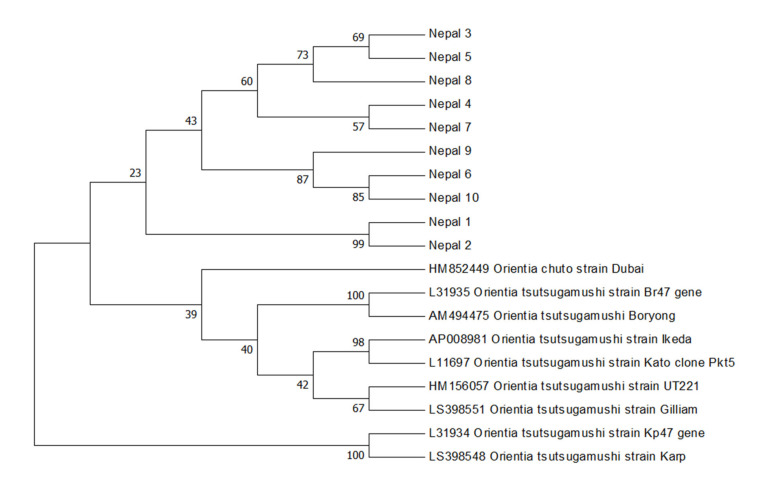
*Orientia tsutsugamushi* 47 kDa gene bootstrap consensus phylogenetic tree. The phylogenetic tree was inferred using the neighbor-joining method. The percentage of replicate trees in which the associated taxa clustered together in the bootstrap test (1000 replicates) are shown next to the branches. The Genbank accession numbers for the *O. tsutsugamushi* strains retrieved are indicated.

A BlastN analysis of Nepal 1–10 47kDa sequences revealed that they shared between 89.7% and 99.3% sequence similarity to the *O. tsutsugamushi* 47 kDa sequences deposited in Genbank. A neighbor-joining bootstrap phylogenetic tree was constructed (Figure 1) to gain insight into the phylogenetic relationships of these novel *O. tsutsugamushi* 47 kDa sequences to those previously described. Neighbor-joining analysis revealed that Nepalese *O. tsutsugamushi* strains clustered together and clustered away from other strains detected in the Middle East and Asia (Figure 1). The 10 sequences of *O. tsutsugamushi* 47 kDa were subsequently submitted to Genbank (Genbank Accession Number #MW657371–MW657380).

**Figure 2:**
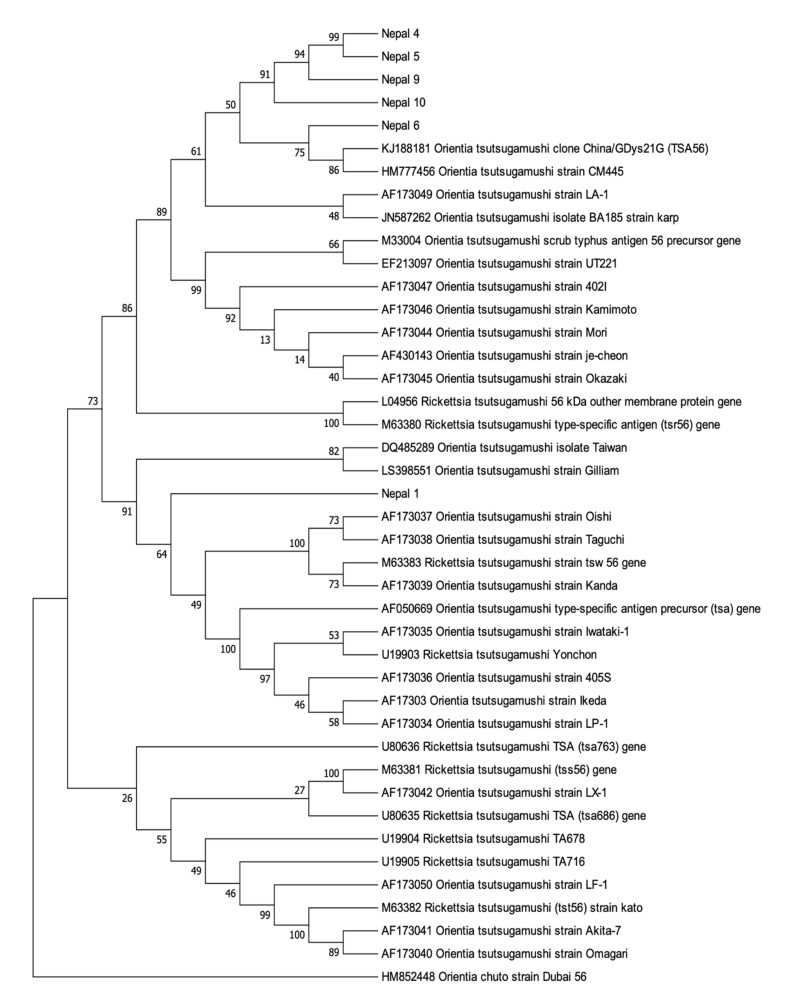
*Orientia tsutsugamushi* 56 kDa gene bootstrap consensus phylogenetic tree. The phylogenetic tree was inferred using the neighbor-joining method. The percentage of replicate trees in which the associated taxa clustered together in the bootstrap test (1000 replicates) are shown next to the branches. The Genbank accession numbers for the *O. tsutsugamushi* strains retrieved are indicated.

Further molecular characterization was obtained by sequencing and phylogenetic analysis of the six 56 kDa gene fragments successfully amplified from Nepal_1, Nepal_4–6, and Nepal_9–10. BlastN searching of Genbank confirmed that these partial Nepalese *O. tsutsugamushi* 56 kDa sequences shared between 93.3% and 99.0% similarity to *O. tsutsugamushi* 56 kDa sequences deposited in Genbank. The results of the phylogenetic analysis of the amplified Nepalese *O. tsutsugamushi* 56 kDa sequences were largely consistent with the analysis of the amplified 47 kDa sequences, demonstrating that the Nepalese strains form a phylogenetically distinct clade from *O. tsutsugamushi* strains detected elsewhere (Figure 2). The exception was the Nepal_1 56 kDa sequence, which was observed forming its own subclade within a phylogenetically distinct clade and away from other Nepalese *O. tsutsugamushi* 56 kDa sequences. The six novel *O. tsutsugamushi* 56 kDa sequences were subsequently submitted to Genbank (Genbank Accession Number #MW657365–MW657370).

## 3. Discussion

Scrub typhus is emerging as a major public health problem in Nepal with reports of its occurrence from several widely reported geographic areas [12,13]. Despite reports of serological evidence of scrub typhus [10], Nepalese molecular surveys have been largely limited to studies of chiggers mite samples and samples from rodents [12]. To date, no molecular studies have been performed to characterize *O. tsutsugamushi*, the causative agent of scrub typhus, from Nepal.

In the current study, we used PCR to screen for the presence of *O. tsutsugamushi*, in blood samples from people with suspected scrub typhus. A prevalence of 29.8% was detected in these samples, confirming the presence of *O. tsutsugamushi* and the suspected diagnosis of scrub typhus in almost a third of these patients. This result is similar to the results of a previous serological survey of blood samples collected from Nepalese patients with acute febrile illness, where 40.3% of blood samples were positive for IgM antibodies against *O. tsutsugamushi* [10]. Notably, the positive patients from this study originated from the Chitwan district in central Nepal. This and adjacent localities were previously shown to be scrub typhus “hotspots” in Nepal, with the Chitwan district itself responsible for 34.4% of the 831 cases of this vector-borne disease in 2016 [12].

Molecular methods such as PCR are the most useful for the diagnosis of scrub typhus in the early stage of disease, before the antibodies are detectable by serological methods. It was demonstrated previously that the performance of PCR-based assays peaked on day 8 of fever, which demonstrated the detection rate to be between 31% and 44%. All DNA-based assays (including isolation) markedly dropped to approximately 20% by day 10 of fever [14]. This reported drop in the PCR detection of *O. tsutsugamushi* in the later stages of scrub typhus fever may explain the absence of *O. tsutsugamushi* DNA in the majority of patients sampled in this study with suspected scrub typhus, while the PCR-positive cases may reflect the potential sampling of individuals during the earlier stages of the clinical presentation of this disease. These results nevertheless confirm that, by broad-range *Rickettsiales* 16S rRNA PCR, *O. tsutsugamushi* DNA can be readily detected in blood samples of Nepalese patients, providing the potential for confirmatory testing if the assays are available for testing.

For the molecular characterization of these Nepalese *O. tsutsugamushi* strains, partial *O. tsutsugamushi* sequences were amplified from the 47 kDa and 56 kDa genes. The 56 kDa type-specific antigen gene consists of unique and cross-reacting epitopes of *Orientia*, contributing to the serological diversity of this species [4]. As already mentioned, sequence analysis of the 56 kDa type-specific antigen is recognized as a valuable and representative method for the genotyping of *O. tsutsugamushi* strains [15,16]. The 47 kDa protein is more conserved than the 56 kDa protein, but retains sufficient diversity to support genetic discrimination [6]. Our analysis of the partial 56 kDa and 47 kDa gene sequences from the Nepalese strains showed that, with the potential exception of Nepal_1, the strains are phylogenetically distinct from those reported in Guangdong, China [17], Thailand [18], and other regions in Asia [19]. For Nepal_1, 47kDa phylogenetic analysis placed this strain amidst the other Nepalese strains, in a subclade with another nearly identical Nepalese *O. tsutsugamushi* 47 kDa sequence (98.7% to Nepal_2; Figure 1). The 56 kDa sequence analysis, however, placed Nepal_1 as a distinct lineage, clustering with a major clade of non-Nepalese *O. tsutsugamushi* strains (Figure 2). A potential explanation for the phylogenetic disparity for Nepal_1 may have to do with observations that the 56 kDa-encoding gene is recombinogenic, with in silico predictions that this *O. tsutsugamushi* gene regularly undergoes recombination events [20] as a part of the broader homologous recombination reported for this pathogen [21]. If this is the correct explanation, it would also imply that there is some level of geographic overlap in hosts that would support the recombination of phylogenetically distinct strains from Nepal and *O. tsutsugamushi* strains from elsewhere in Asia.

## 4. Materials and Methods

### 4.1. Study Population

A cross-sectional descriptive study was conducted among patients with acute febrile illness with suspected scrub typhus from the Chitwan district in central Nepal for a duration of eight months, between April 2018 and November 2018. Cases of acute fever without localizing features that had more than four days duration were included in the study, following the exclusion of confirmed cases of other febrile illnesses including malaria, dengue, leptospirosis, and enteric diseases.

Blood samples were collected from the patients suspected of scrub typhus presenting with acute febrile illness in EDTA blood tubes. The buffy coat was separated and stored at −80 °C. During the time of admission, a structured questionnaire was completed to gain data, such as demographic variables, to analyze the disease epidemiology.

### 4.2. Rickettsia PCR Screening and Sequencing

DNA was extracted from the buffy coat using a HiYield^TM^ DNA Mini Kit (YGB 300, Real Genomics, Taipei, Taiwan), and tested using rickettsial qPCR procedures established at the Australian Rickettsial Reference Laboratory (ARRL, Geelong, Australia) [22]. Briefly, the qPCR assay targeted the *O. tsutsugamushi* 16S rRNA gene using primers 16S rRNA-F (5′-CTTATTTGCCAGCGGGTAATGC-3′) and 16S rRNA-R (5′-GGGCCATGATGACTTGACCTC-3′), and the probe 16S rRNA-Probe (5′-FAM-CCCACCTTCCTCCGGCTTAGCACCH-BQ10-3′). Each reaction contained 200 nM of each primer and probe, 2 × Platinum qPCR Super Mix-UDG Mastermix (Invitrogen, Melbourne, Australia), 5 mM MgCl_2_, and a DNA template to a total reaction volume of 25 µL. The reactions were performed and analyzed using the RotorGene 3000 (Corbett Research, Sydney, Australia) with positive and negative controls. The cycling parameters included an initial holding temperature of 50 °C for 3 min, followed by 95 °C for 5 min and 65 cycles of 95 °C for 20 s and 60 °C for 40 s. Any samples with Ct values of <35 were deemed positive. Ct values between 35 and 40 were considered equivocal and were repeated for confirmatory testing. Ct values <40 were considered negative. 

For rickettsial PCR-positive specimens, a conventional PCR targeting the 56 kDa type-specific gene was performed using the forward (5′-TACATTAGCTGCAGGTATGACA-3′) and reverse primers (5′-CCAGCATAATTCTTTAACCAAG-3′) (Invitrogen, Melbourne, Australia), as previously described [23]. Additional sequence information for each rickettsial-positive specimen was obtained by PCR amplification of the 47 kDa HtrA sequences using primers targeting members of the genus *Orientia*, as previously described [6]. Positive (*Orientia tsutsugamushi* Karp and Kato strains) and negative (dH_2_O) controls were included with every run for both PCRs.

Amplicon sequencing of the 56 kDa and 47 kDa PCR products was performed at Macrogen (Seoul, Korea). Sequences obtained were compared with sequences available by BlastN analysis (http://blast.ncbi.nlm.nih.gov accessed on 15 February 2021). The relationship of the Nepalese phylogenetic analysis was performed by the construction of the phylogenetic trees using the amplified 56 kDa and 47 kDa partial gene sequences separately. A phylogenetic tree was constructed from *O. tsutsugamushi* strains obtained from Genbank using the neighbor-joining method [24], as presented in MEGA 7 [25].

## 5. Conclusions

This study provides concrete molecular evidence for the presence of *O. tsutsugamushi* as the causative agent of scrub typhus in Nepal. This demonstration may help in the implementation of rapid diagnostics and early treatment of this disease in this region. Conventional PCR and the subsequent sequencing of the 56 kDa and 47 kDa antigens of this pathogen reveal that Nepalese strains are largely genetically distinct from those reported elsewhere in Asia, although we detected at least one strain that may share evolutionary ancestry with non-Nepalese *O. tsutsugamushi* strains through a process of homologous recombination. Knowledge of the circulating *O. tsutsugamushi* antigenic diversity of Nepalese strains may be important in the development of future diagnostic tests and vaccines that support efforts to control this important neglected tropical disease in this country.

## Data Availability

The data presented in this study are openly available in GeneBank (for accession numbers see above).
